# Safety and efficacy of immune checkpoint inhibitor rechallenge in advanced non-small cell lung cancer: a retrospective study

**DOI:** 10.1038/s41598-024-52034-2

**Published:** 2024-01-28

**Authors:** Jia Feng, Xinyi Chen, Jiayan Wei, Yiming Weng, Jingsong Wang, Tong Wang, Qibin Song, Peng Min

**Affiliations:** Cancer Center, Renmin Hospital of Wuhan University, Wuhan, 430060 China

**Keywords:** Cancer, Oncology

## Abstract

We conducted a retrospective study to evaluate the efficacy of immune checkpoint inhibitor (ICI) rechallenge in patients with advanced non-small cell lung cancer (NSCLC). The study included 111 patients who had previously received ICI therapy and experienced disease progression. The primary endpoints assessed were overall survival (OS), progression-free survival (PFS), and objective response rate (ORR). Our findings revealed that the ICI rechallenge showed promising results in improving patient outcomes. OS (r) is the time from rechallenging with immune checkpoint inhibitors to the last follow-up or death from any cause. The median OS (r) was 14.3 months (95% CI 11.3–17.3 months), with a median PFS (r) of 5.9 months (95% CI 4.1–7.7 months). The ORR was 17.1%; the DCR was 82.3%. Subgroup analysis demonstrated that patients without brain or liver metastases had a longer OS (r) compared to those with metastases (21.6 vs. 13.8 months, χ^2^ = 3.873, *P* = 0.046; 20.8 vs. 9.1 months, χ^2^ = 10.733, *P* = 0.001, respectively). Moreover, patients without driver gene mutations exhibited significantly longer OS than those with mutations or wild-type patients (22.9 vs. 16.1 vs. 7.5 months, χ^2^ = 10.710, *P* = 0.005). Notably, patients who switched to a different ICI during the rechallenge had shorter OS than those who did not change medications (10.4 vs. 21.1 months, χ^2^ = 9.014, *P* = 0.003). The incidence of immune-related adverse events did not significantly differ between the two treatment phases. These findings suggest that ICI rechallenge may be a viable therapeutic strategy for select NSCLC patients. Further prospective studies are needed to validate these results and guide treatment decisions for advanced NSCLC.

## Introduction

Non-small cell lung cancer (NSCLC) is one of the most prevalent types of cancer worldwide and remains a significant cause of cancer-related deaths^1^. Despite advancements in treatment options, including targeted therapies and chemotherapy, the prognosis for advanced NSCLC patients has been historically poor^2^. The emergence of immune checkpoint inhibitors (ICI) has revolutionized the treatment landscape for NSCLC and significantly improved the prognosis of patients with lung cancer.

ICIs enhance the body's immune response against cancer cells, enabling the immune system to recognize and attack tumor cells more effectively. Consequently, ICI therapy has led to significant improvements in overall survival (OS) and progression-free survival (PFS) in a subset of NSCLC patients^3^. Despite the initial success of ICI therapy, a considerable number of patients eventually experience disease progression or develop resistance to treatment. For patients with disease progression after using ICI, only a limited number of treatment options are available. In clinical practice, systemic treatments, including targeted therapy and chemotherapy, are commonly used, but their efficacy is limited^4,5^. In such cases, the concept of ICI rechallenge, where patients are re-exposed to ICIs after disease progression, has gained increasing attention. Several studies have suggested that ICI rechallenge may lead to additional clinical benefits, including prolonged PFS and improved response rates, in select NSCLC patients^6–8^.

However, the efficacy and safety of ICI rechallenge in advanced NSCLC remain subjects of ongoing research and debate^9,10^. This retrospective study aims to analyze the safety and effectiveness of ICI rechallenge and investigate factors associated with treatment response. By examining the outcomes of the ICI rechallenge, we hope to gain valuable insights into its potential benefits and limitations.

## Materials and methods

### Patients

The study included 111 patients with advanced non-small cell lung cancer who were retreated with immunotherapy at Renmin Hospital of Wuhan University between 1st September 2018 and 30th June 2022. The following are the inclusion criteria: ① At the start of the first ICI, the pathology diagnosis is advanced-stage NSCLC (according to the 8th edition TNM staging, diagnosed as stage III B to IV); ② The ICI rechallenge was started because of disease progression; ③ All patients were in Eastern Cooperative Oncology Group-Performance Status (ECOG-PS) ≤ 3 Points; ④ At least one primary or metastatic lesion that can be measured or evaluated by imaging data, as well as relevant imaging data for measurement and evaluation; ⑤ Clinical and pathological data are complete. The exclusion criteria were as follows: ① The presence of concurrent other primary tumors; ② Patients who discontinued immunotherapy due to toxicity.

## Data collection

Patients’ clinical and survival data were collected retrospectively, including age, gender, histological type, clinical stage, metastatic site, PD-L1 expression, driver gene mutation, and treatment information. The PD-L1 Tumor Proportion Score (TPS) assesses PD-L1 expression, representing the percentage of PD-L1-positive tumor cells displaying partial or complete membrane staining across the entire tumor slide. We classified PD-L1 expression into three levels: PD-L1 TPS < 1%, PD-L1 TPS 1–49%, and PD-L1 TPS ≥ 50%. The severity of immune-related adverse events (IRAEs) was evaluated using the Common Terminology Criteria for Adverse Events version 4.0.

### Follow-up

Follow-up was based on inpatient system review or telephone follow-up until May 10, 2023. Patients were assessed for efficacy every two treatment cycles until death or the end of the study. The best overall response (BOR) assessment was performed based on the Response Evaluation Criteria in Solid Tumors Version 1.1 (RECIST v.1.1), which included complete response (CR), partial response (PR), stable disease (SD), and progressive disease (PD). Objective response rate (ORR) was calculated as a percentage of patients who obtained CR or PR. Disease control rate (DCR) was the percentage of patients who achieved CR, PR, or SD. PFS (i) is the time from initiating immune checkpoint inhibitor therapy to the first occurrence of disease progression. PFS (r) is the time from rechallenging with immune checkpoint inhibitors to subsequent disease progression. OS (i) represents the time from initiating immune checkpoint inhibitor therapy to the last follow-up or death from any cause. OS (r) is the time from rechallenge with immune checkpoint inhibitors to the last follow-up or death from any reason. (PFS stands for Progression-Free Survival, OS stands for Overall Survival, and (i) and (r) denote the initial and rechallenge settings, respectively.)

### Statistical analysis

We estimated the survival curves of OS(i), OS(r), PFS(i), and PFS(r) using the Kaplan–Meier method, and differences were compared using the log-rank test. The hazard ratios (HRs) and their 95% confidence intervals (CIs) were estimated using the Cox proportional hazards model in multivariate analyses. We used SPSS 26.0 and Prism 9.5.0 for all statistical analyses.

### Ethics statement

The inclusion of patients complies with the Helsinki Declaration and has been approved by the Clinical Research Ethics Committee of Wuhan University People's Hospital. Informed consent was obtained from all subjects involved in the study or from their legal guardian(s) before their participation, and all patient data collected during follow-up adhered to relevant data protection and privacy regulations.

## Results

### Patients characteristics

One hundred eleven patients with advanced non-small cell lung cancer (NSCLC) were included in the present study. The demographics and clinicopathological characteristics of patients are shown in Table 1. The median age was 63 years (40–81 years), with a higher proportion of male patients (89 cases, 80.2%); 43 of the individuals had a smoking history. Adenocarcinoma was the predominant histological subtype observed in this study. Most patients (98 cases, 88.3%) were diagnosed with stage IV at the initiation of immunotherapy rechallenge. Among the cohort, 71 patients underwent driver gene testing, of which 28 cases exhibited sensitive mutations, including EGFR sensitive mutations, ALK fusion-positive, ROS1 fusion-positive, MET exon 14 skipping mutations, KRAS G12C/D/V mutations, and Her-2 mutations. Six cases were identified as wild-type. About half (43%) of the patients in this research had PD-L1 immunohistochemistry (IHC) testing done. Among them, 53.3% of the patients were found to have negative PD-L1 expression (TPS < 1%), while 38.3% of patients were PD-L1 highly expressed (TPS ≥ 50%). The majority of patients (95 cases, 85.6%) had distant metastases at the start of immunotherapy rechallenge, among whom 33/95 (34.7%) were in M1b stage (distant metastases outside the lung/pleura) and 37/95 (38.9%) were in M1c stage (multiple metastases).

**Table 1:** Demographics and clinicopathological characteristics of patients (n = 111).

Characteristics	Number of patients (%)
Gender	
Male	89 (80.2%)
Female	22 (19.8%)
Age	
≤ 63	63 (56.8%)
> 63	48 (43.2%)
Smoking status	
Never-smokers	68 (61.3%)
Former or current smokers	43 (38.7%)
Histological subtype	
Adenocarcinoma	70 (63.1%)
Squamous carcinoma	41 (36.9%)
Clinical stage at ICI rechallenge initiation	
IIIB–C	13 (11.7%)
IV	98 (88.3%)
PD-L1 expression	
Number tested	47 (42.3%)
TPS < 1%	20/47 (53.3%)
1% ≤ TPS < 50%	9/47 (19.1%)
TPS ≥ 50%	18/47 (38.3%)
Unknown	64 (57.7%)
Driver genes	
Number tested	71 (64.0%)
Negative	37/71 (52.1%)
Sensitive mutations	28/71 (39.4%)
Wildtype	6/71 (8.5%)
Unknown	40 (36.0%)
M Staging at ICI rechallenge initiation	
0	16 (14.4%)
1a	25 (22.5%)
1b	33 (29.7%)
1c	37 (33.3%)
Metastatic sites at ICI rechallenge initiation	
Brain	33(29.7%)
Liver	15(13.5%)
Bone	43(38.7%)

## Immunotherapy regimens

In the initial immunotherapy, most patients (69 cases, 62.2%) received immune checkpoint inhibitors (ICIs) in combination with chemotherapy. The most common chemotherapy regimens included paclitaxel/nab-paclitaxel or pemetrexed combined with platinum-based drugs. Among the patients, 49 (44.1%) received initial immunotherapy as first-line treatment, while 35 (31.5%) received it as second-line treatment. The remaining patients had received immunotherapy as a third-line or later treatment. After the initial immunotherapy progressed, only 20 cases (18.0%) switched to a different ICI. The most common rechallenge regimen was ICI combined with chemotherapy and/or anti-angiogenic therapy (67 cases, 60.1%) (Table 2).

**Table 2:** Data about patients that received initial immunotherapy and ICI rechallenge.

	Number of patients
Initial immunotherapy regimen	
ICI monotherapy	11
Combination chemotherapy	69
paclitaxel/nab-paclitaxel ± platinum	30
docetaxel ± platinum	5
pemetrexed ± platinum	28
gemcitabine ± platinum	5
tegafur	1
Combination angiogenesis inhibitor	6
bevacizumab	2
anlotinib	2
endostar	1
apatinib	1
Combination chemotherapy + angiogenesis inhibitor	25
paclitaxel/nab-paclitaxel ± platinum + endostar	5
nab-paclitaxel ± platinum + bevacizumab	3
nab-paclitaxel + tegafur + apatinib	1
docetaxel ± tegafur + anlotinib	3
pemetrexed ± platinum + endostar	5
pemetrexed ± platinum + bevacizumab	5
pemetrexed + anlotinib	2
etoposid + platinum + endostar	1
Line of initial immunotherapy	
1	49
2	35
≥ 3	27
ICI rechallenge regimen	
ICI monotherapy	15
Combination chemotherapy	30
paclitaxel/nab-paclitaxel ± platinum	13
docetaxel ± platinum	5
pemetrexed ± platinum	8
gemcitabine ± platinum	2
tegafur	2
Combination angiogenesis inhibitor	29
bevacizumab	5
anlotinib	18
endostar	2
apatinib	1
lenvatinib	3
Combination chemotherapy + angiogenesis inhibitor	37
paclitaxel/nab-paclitaxel ± platinum + endostar	4
nab-paclitaxel ± platinum + bevacizumab	8
nab-paclitaxel + tegafur + anlotinib	1
docetaxel ± platinum + anlotinib	3
pemetrexed ± platinum + endostar	4
pemetrexed ± platinum + bevacizumab	4
pemetrexed ± platinum + anlotinib	7
gemcitabine + bevacizumab	3
tegafur + anlotinib	3
Rechallenge with the same ICI	
Yes	20
No	91

## Efficacy of ICI treatments

**Figure 1:**
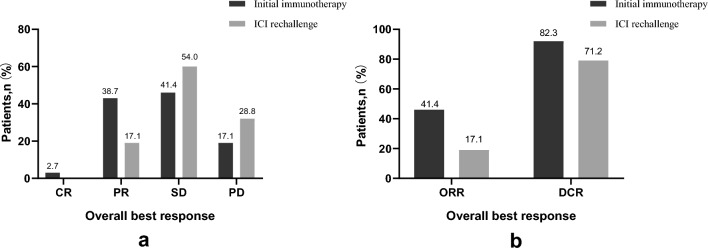
Frequency of the best overall response to ICI treatment. (**a**) Tumor responses in initial immunotherapy and ICI rechallenge. (b) ORR and DCR in initial immunotherapy and ICI rechallenge.

In the initial immunotherapy, the median PFS(i) and OS(i) were 6.6 months (95% CI 5.3–7.9 months) and 25.0 months (95% CI 22.0–28.0 months), respectively. Among them, 3 cases (2.7%), 43 cases (38.7%), 46 patients (41.4%), and 19 cases (17.1%) achieved CR, PR, SD, and PD, respectively. The ORR and DCR were 41.4% and 82.3%, respectively. In the ICI rechallenge, 85 patients progressed with a median PFS(r) of 5.9 months (95% CI 4.1–7.7 months) and a median OS(r) of 14.3 months (95% CI 11.3–17.3 months). In total, 19 cases (17.1%), 60 cases (54%), and 32 cases (28.8%) achieved PR, SD, and PD, respectively. The ORR and DCR were 17.1% and 71.2%, respectively (Fig. 1a, b). Compared to the initial ICI treatment, ICI rechallenge showed lower ORR and DCR (ORR: χ^2^ = 15.859, *P* < 0.001; DCR: χ^2^ = 4.302, *P* = 0.038).

Further analysis revealed that the efficacy of different ICI rechallenge regimens may vary. The ORR for the ICI monotherapy group, ICI combined with chemotherapy group, ICI combined with anti-angiogenic therapy group, and ICI combined with chemotherapy and anti-angiogenic therapy group were 20%, 10%, 20.7%, and 18.9%, respectively. The DCR for the respective groups were 73.3%, 66.7%, 55.2%, and 86.5% (Table 3). The ICI combined with chemotherapy and anti-angiogenic therapy group showed a higher DCR than the other groups (χ^2^ = 8.178, *P* = 0.042).

**Table 3:** Efficacy evaluation in different subgroups.

Group	PR	SD	PD	ORR	DCR
Total	19 (17.1%)	60 (54%)	32 (28.8%)	19 (17.1%)	92 (71.2%)
ICI rechallenge regimen					
ICI monotherapy	3 (20.0%)	8 (53.3%)	4 (26.7%)	3 (20.0%)	11 (73.3%)
Combination chemotherapy	3 (10.0%)	17 (56.7%)	10 (33.3%)	3 (10.0%)	20 (66.7%)
Combination angiogenesis inhibitor	6 (20.7%)	10 (34.5%)	13 (44.8%)	6 (20.7%)	16 (55.2%)
Combination chemotherapy + angiogenesis inhibitor	7 (18.9%)	25 (67.6%)	5 (13.5%)	7 (18.9%)	32 (86.5%)

## Progression-free survival at ICI rechallenge

**Figure 2:**
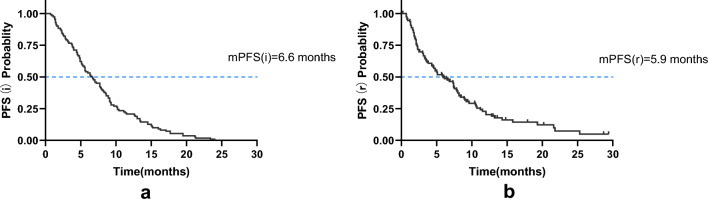
Kaplan–Meier survival curves for PFS(i) and PFS(r).

**Figure 3:**
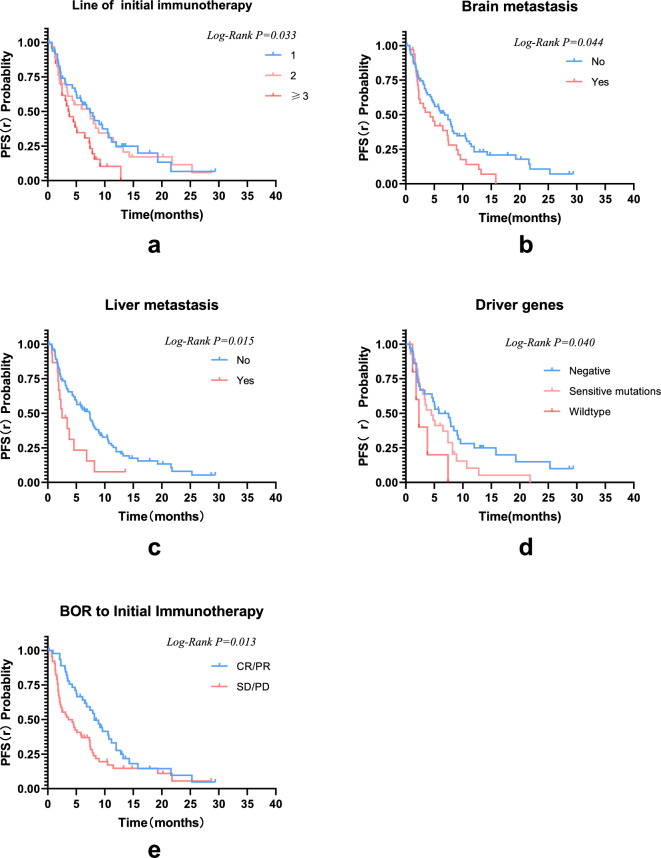
Kaplan–Meier curves depicting the PFS(r) in relation to various factors: (**a**) line of initial immunotherapy, (**b**) presence or absence of brain metastasis, (**c**) presence or absence of liver metastasis, (**d**) driver gene mutation status, and (**e**) BOR to initial immunotherapy.

**Figure 4:**
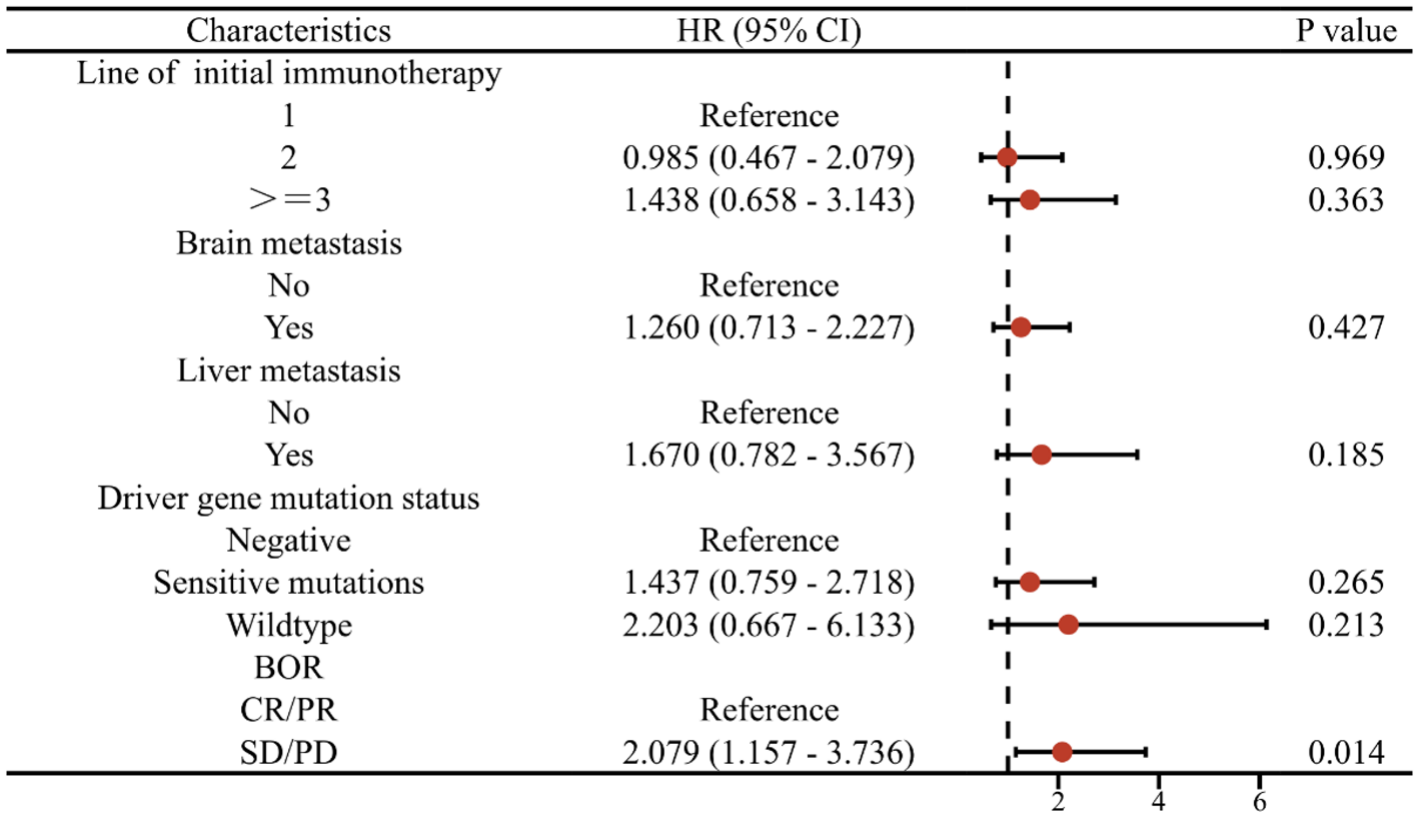
Forest plot of PFS(r) in patients with different line of initial immunotherapy, presence or absence of brain metastasis, presence or absence of liver metastasis, driver gene mutation status, and the BOR to initial immunotherapy.

In the initial immunotherapy, the median PFS (i) was 6.6 months (95% Cl 5.3–7.9 months). In the ICI rechallenge, 85 patients progressed with a median PFS(r) of 5.9 months (95% CI 4.1–7.7 months) (Fig. 2). In subgroup analysis, patient PFS(r) was not associated with gender, age, pathological type, smoking status, switch of immunotherapy agents, PD-L1 TPS, rechallenge regimens、bone metastasis, or M staging (*P* > 0.05). A tendency towards an extended PFS(r) was observed in patients who received 1 or 2 lines of initial immunotherapy, had no brain metastasis, no liver metastasis, no driver gene mutations, and achieved a CR or PR as the BOR to the initial immunotherapy. In patients receiving first-line initial immunotherapy, the PFS (r) is superior to that in the second-line (9.6 months vs. 9.1 months) and even more prominent when compared to the third-line or above (9.6 months vs. 5.0 months), with a statistically significant difference (χ^2^ = 6.824, *P* = 0.033). Patients without brain metastasis demonstrated a longer PFS(r) than those with brain metastasis (9.5 vs. 5.8 months, χ^2^ = 4.042, *P* = 0.044). Similarly, patients without liver metastasis exhibited a longer PFS(r) than those with liver metastasis (8.9 vs. 4.1 months, χ^2^ = 5.863, *P* = 0.015). It was observed that patients without driver gene mutations had a significantly longer PFS(r) compared to patients with mutations and wild-type patients (9.5 vs. 5.9 vs. 3.3 months, χ^2^ = 6.441, *P* = 0.040). Additionally, those who acquired a CR/PR as the BOR to the initial ICI had a longer PFS(r) than patients with SD/PD (10.2 vs. 7.0 months, χ^2^ = 6.225, *P* = 0.013) (Fig. 3). Statistically significant factors were incorporated in the multivariable Cox regression model, revealing that the BOR to the initial ICI was an independent prognostic factor for PFS(r) (Fig. 4).

## Overall survival

**Figure 5:**
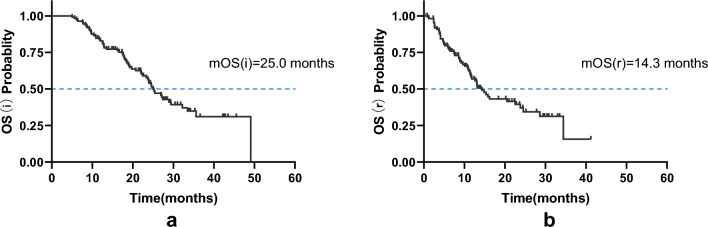
Kaplan–Meier survival curves for OS(i) and OS(r).

**Figure 6:**
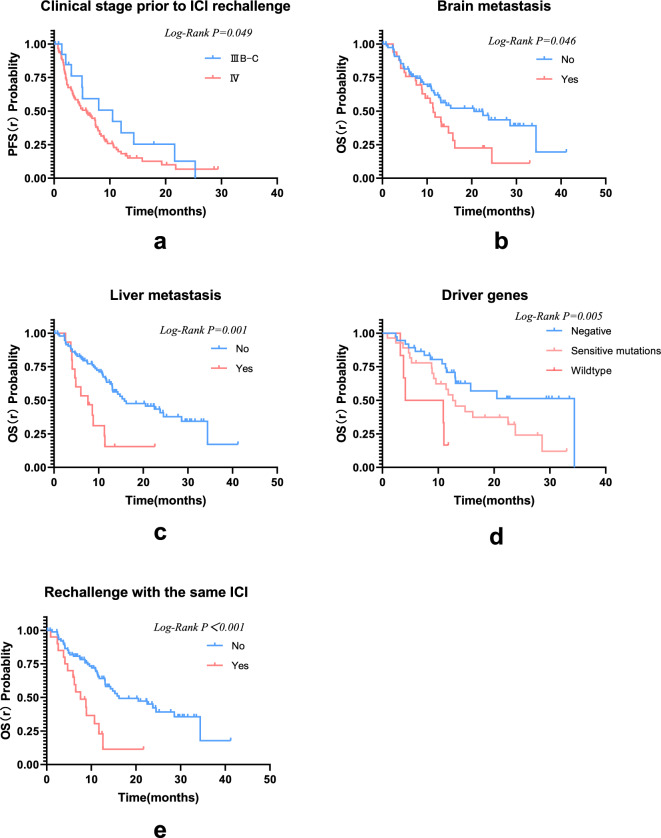
Kaplan–Meier curves depicting the OS(r) in relation to various factors: (**a**) clinical stage prior to ICI rechallenge, (**b**) presence or absence of brain metastasis, (**c**) presence or absence of liver metastasis, (**d**) driver gene mutation status, and (**e**) the same ICI rechallenge or not.

**Figure 7:**
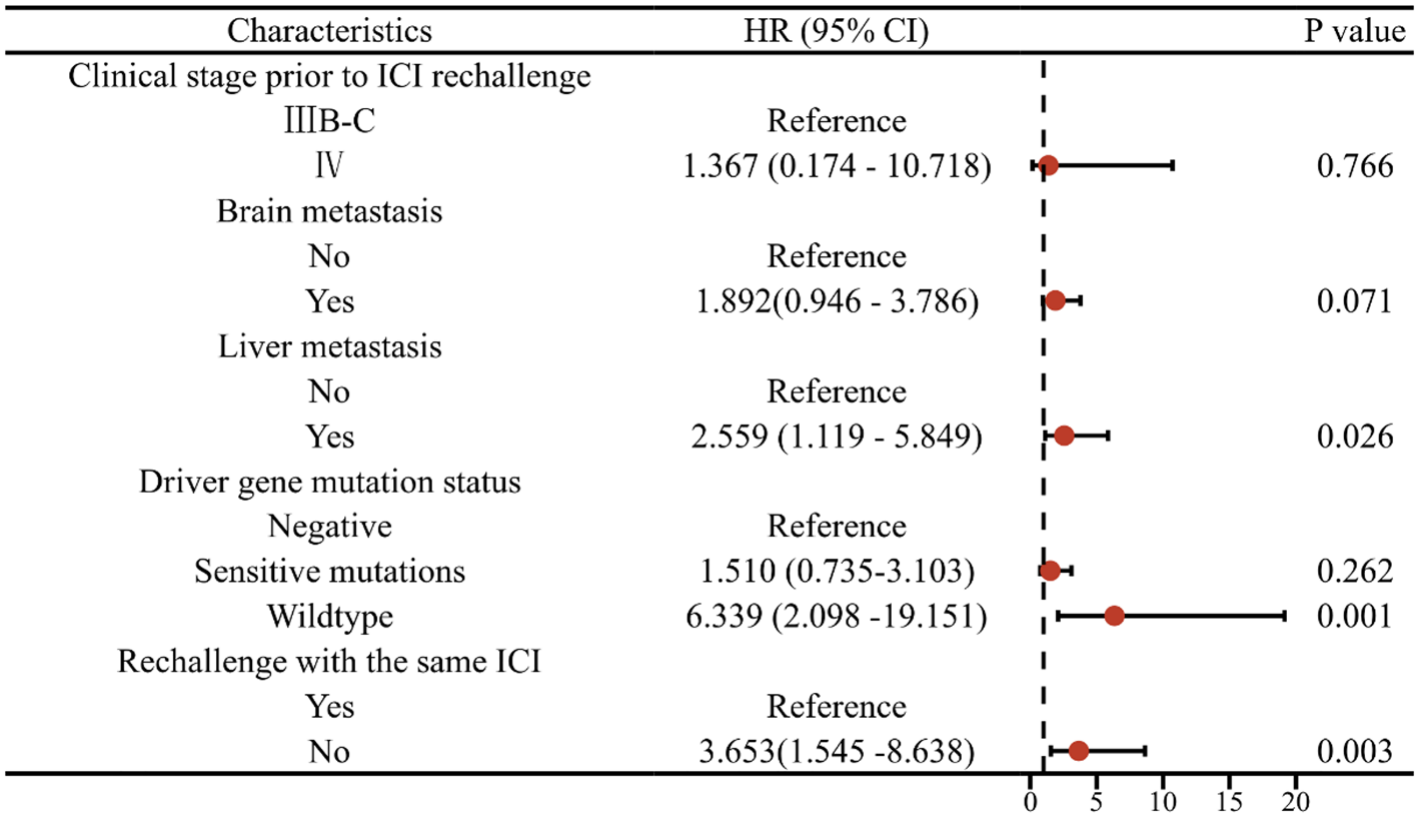
Forest plot showing OS(r) in relation to clinical stage, brain metastasis, liver metastasis, driver gene mutation status, and same ICI rechallenge.

During follow-up, 60 patients (54.1%) had died, the median OS (i) was 25 months (95% CI 22.0–28.0 months), and median OS (r) was 14.3 months (95% CI 11.3–17.3 months)(Fig. 5). Our analysis revealed several important observations. Patients in stage III had a significant OS (r) compared to those in stage IV (28.2 vs. 17.3 months, χ^2^ = 3.889, *P* = 0.049). Patients without brain metastasis or liver metastasis demonstrated more prolonged overall survival (r) with 21.6 versus 13.8 months (χ^2^ = 3.873, *P* = 0.046) and 20.8 versus 9.1 months (χ^2^ = 10.733, *P* = 0.001), respectively. Additionally, patients without driver gene mutations had a substantially longer OS (r) than those with mutations or wild-type patients (22.9 vs. 16.1 vs. 7.5 months, χ^2^ = 10.710, *P* = 0.005). Lastly, patients who switched to a different ICI during the rechallenge had a shorter OS (r) compared to those who did not change medications (10.4 vs. 21.1 months, χ^2^ = 9.014, *P* = 0.003) (Fig. 6). The multivariable Cox regression analysis revealed that clinical stage, brain metastasis, liver metastasis, driver gene mutation status, and same ICI rechallenge were independent prognostic factors for OS(r) (Fig. 7).

## Immune-related adverse events

Immune-related adverse events (irAEs). The initial immunotherapy and rechallenge are listed in Table 4. During the initial immunotherapy, the overall incidence of irAEs of any grade was 45.0%. The most commonly observed irAEs were rash/dermatitis, pneumonitis, and hypothyroidism. Grade 3 or higher irAEs were observed in a small subset of patients (15 cases, 13.5%). During the ICI rechallenge phase, the incidence of irAEs of all grades was 37.8%, with Grade 3 or higher irAEs occurring in 18.0% of patients. There was no significant difference in the incidence of all-grade and grade 3 or higher immune-related adverse events (irAEs) between the initial ICI treatment and ICI rechallenge phases (all grades: χ^2^ = 1.188, *P* = 0.276; grade 3 or higher grades: χ^2^ = 0.848, *P* = 0.357). Furthermore, the two phases observed no significant differences in adverse events (*P* > 0.05). Notably, no treatment-related deaths were reported throughout the initial immunotherapy and ICI rechallenge phases. Additionally, no instances occurred where the treatment had to be discontinued due to adverse events.

**Table 4:** Immune-related adverse events (irAEs) during initial immunotherapy and rechallenge.

Immune-related adverse events	Any grade, n (%)		*P* value	≥ Grade 3, n (%)		*P* value
	Initial immunotherapy	ICI rechallenge		Initial immunotherapy	ICI rechallenge	
Rash/dermatitis	11 (9.9%)	7 (9.9%)	0.325	4 (3.6%)	3 (2.7%)	0.701
CCEP	3 (2.7%)	1 (0.9%)	0.313	1 (0.9%)	0	0.316
Hypothyroidism	7 (6.3%)	4 (3.6%)	0.354	1 (0.9%)	2 (1.8%)	0.561
Increased alanine aminotransferase	6 (5.4%)	3 (2.7%)	0.307	1 (0.9%)	2 (1.8%)	0.561
Diarrhea/colitis	5 (4.5%)	8 (7.2%)	0.391	3 (2.7%)	5 (4.5%)	0.471
Pneumonitis	8 (7.2%)	9 (8.1%)	0.801	2 (1.8%)	4 (3.6%)	0.408
Arthralgia	3 (2.7%)	6 (5.4%)	0.307	0	2 (1.8%)	0.155
Myocarditis	2 (1.8%)	1 (0.9%)	0.561	0	0	1.000
Renal insufficiency	5 (4.5%)	3 (2.7%)	0.471	3 (2.7%)	2 (1.8%)	0.651

*CCEP* cutaneous capillary endothelial proliferation; Grade according to the Common Terminology Criteria for Adverse Events version 4.0

## Discussion

Currently, the efficacy of ICI rechallenge remains controversial. Some studies suggest that ICI-refractory NSCLC may have limited benefits from ICI rechallenge. For instance, Fujita et al. reported that in 12 NSCLC patients who experienced nivolumab resistance, the ORR, DCR, and PFS of pembrolizumab rechallenge were 8.3%, 41.7%, and 3.1 months, respectively^11^. Fujita also reported that the ORR, DCR, and PFS of atezolizumab rechallenge after refractory ICI treatment in NSCLC patients were 0%, 38.9%, and 2.9 months, respectively^9^. Watanabe et al. reported an ORR of 7.1%, a DCR of 21.4%, and a PFS of 1.6 months in 14 ICI-refractory patients who underwent ICI rechallenge^12^. In our study of 111 patients with advanced NSCLC, ICI rechallenge resulted in an ORR of 17.1%, a DCR of 71.2%, and median PFS and OS values of 5.9 months and 14.3 months, respectively. In comparison to the initial ICI treatment, ICI rechallenge demonstrated a lower ORR and DCR (ORR: χ^2^ = 15.859, *P* < 0.001; DCR: χ^2^ = 4.302, *P* = 0.038), indicating limited clinical benefits compared to the initial ICI treatment. However, the data showed that PFS and OS were superior to NSCLC patients who received salvage chemotherapy after failed immunotherapy^13–15^.

The most common rechallenge regimen was ICI combined with chemotherapy and/or anti-angiogenic therapy. Further analysis revealed that the ICI combined with chemotherapy and anti-angiogenic therapy group showed a higher DCR than the other groups (χ^2^ = 8.178, *P* = 0.042). Anti-angiogenic drugs alter tumor vasculature and promote immune cell infiltration, but they also improve immune effector cell cytotoxicity, promoting their delivery to tumor locations^16,17^. This enhances the efficacy of immunotherapy. In the IMpower150 study, the combination of atezolizumab and bevacizumab significantly increased the PFS of patients^18^. Consistent with previous reports in melanoma patients^19,20^, our study reveals a clear association between the response to initial ICI treatment and the PFS during ICI rechallenge in NSCLC patients. Importantly, we identified the BOR to the initial ICI treatment as an independent prognostic factor for PFS(r). These findings align with the study conducted by Akamatsu et al., which also demonstrated limited overall efficacy of ICI rechallenge (ORR: 8.5%, median PFS: 2.6 months). However, it is worth noting that a subset of five responders exhibited a significantly prolonged median PFS of 11.1 months^21^

At the time of the ICI rechallenge, the patients included in our study had 35 out of 111 individuals who had previously received two lines of immunotherapy. In patients receiving first-line initial immunotherapy, the PFS (r) was superior to second-line and third-line (9.6 vs. 9.1 vs. 5.0 months) (χ^2^ = 6.824, *P* = 0.033). This result is also consistent with the facts. We found that patients in stage III had a significantly longer OS (r) than stage IV patients (28.2 vs. 17.3 months, χ^2^ = 3.889, *P* = 0.049). This finding aligns with the notion that early-stage disease is associated with better prognoses and higher chances of treatment success. The cancer stage at the time of ICI rechallenge may influence the effectiveness of the treatment and subsequent survival outcomes. Additionally, patients without brain and liver metastasis exhibited longer OS (r) than their counterparts with metastases in these organs. Another cohort study has suggested that patients who did not have brain or liver metastasis and achieved a PR to the initial ICI treatment were more likely to benefit from ICI rechallenge^22^

The observed longer OS (r) in patients without driver gene mutations compared to those with mutations or wild-type patients (22.9 vs. 16.1 vs. 7.5 months, χ^2^ = 10.710, *P* = 0.005) may be attributed to the fact that driver gene-positive patients received ICIs as a later-line treatment after developing resistance to targeted therapies. Previous studies have indicated that immune checkpoint inhibitors (ICIs) do not improve overall survival (OS) in patients with EGFR-mutated NSCLC^23–26^. A meta-analysis revealed that patients with EGFR mutations did not derive benefit from PD-1/PD-L1 blockade therapy. OS and PFS were lower in EGFR-mutated patients than those without mutations^27^.

Lung cancer in never smokers (LCINS) demonstrates poor response to immune checkpoint inhibitors (ICI)^28^. LCINS accounted for 61.3% of the cases in this study. However, we did not find differences in PFS (i), PFS (r), OS (i), and OS (r) between the Never-smokers group and the Former or current smokers group. Gainor et al. observed overall response rates of (27% vs. 40% vs. 40%) progression-free survival of (3.0 vs. 4.0 vs. 5.4 months) and duration of response (6.9 vs. 10.8 vs. 17.8 months) in smokers, never smokers, and light smokers, respectively^29^. Popat et al. conducted a retrospective cohort study of 1160 patients treated with single-agent ICI, including 91 never smokers. Former smokers exhibited an OS time nearly double that of never smokers (12.8 months and 6.5 months, respectively). We did not observe this outcome, possibly due to the limited sample size.

Furthermore, we noted that patients who switched to a different ICI during the rechallenge had shorter OS (r) than those who did not change medications (10.4 vs. 21.1 months, χ^2^ = 9.014, *P* = 0.003). This finding suggests that maintaining continuity in ICI treatment may be associated with better outcomes. Previous studies have demonstrated no significant difference in ORR and DCR between ICI rechallenge and previous ICI treatment of the same type (*P* > 0.05). However, compared to the initial treatment, different types of ICIs are associated with a decrease in ORR and DCR (ORR: OR, 0.09; 95% CI 0.02–0.34; *P* < 0.05; I^2^ = 0%) (DCR: OR, 0.35; 95% CI 0.18–0.67; *P* < 0.05; I^2^ = 0%)^30^. Plazy et al.^8^ also reported that patients with a longer duration of initial ICI treatment and those who switched to the same type of ICI rather than anti-PD-1 or anti-PD-L1 agents derived more benefit from ICI rechallenge. However, some studies have reported similar ORR when ICIs are switched to another type or combined with other drugs compared to initial immunotherapy^20^.

Regarding safety, the initial ICI treatment and ICI rechallenge demonstrated acceptable safety profiles, with no treatment-related deaths reported and no cases requiring treatment discontinuation due to adverse events. The incidence of immune-related adverse events (irAEs) did not significantly differ between the two treatment phases. This indicates that ICI rechallenge can be safely administered to patients who have previously received ICI therapy, without an increased risk of severe or life-threatening adverse events. The higher incidence of Grade 3 or higher irAEs in the rechallenge group suggests that caution should be exercised when considering ICI rechallenge in patients who have previously experienced significant immune-related toxicities.

We acknowledge the limitations of our study. The retrospective nature of the analysis and the relatively small sample size may have introduced biases and limited the generalizability of the findings. Notably, over 50% of patients did not undergo PD-L1 testing, which prompts a discussion regarding the potential impact of PD-L1 expression on treatment outcomes. Additionally, the heterogeneity of treatment regimens and the lack of standardized rechallenge protocols may have influenced the results and safety profiles. In conclusion, our study provides valuable insights into the efficacy and safety of ICI rechallenge in patients with advanced NSCLC. Future prospective studies with larger sample sizes and standardized protocols are warranted to validate these findings further and optimize the clinical use of ICI rechallenge in this patient population.

## Conclusion

ICI rechallenge demonstrates promising efficacy in patients with advanced NSCLC, particularly those without brain or liver metastasis and driver gene mutations. However, careful consideration is needed when switching to different ICIs during rechallenge. Further prospective studies are warranted to validate these findings and guide clinical decision-making for optimal treatment strategies in advanced NSCLC patients.

## Data Availability

Specific inquires for our data and analyses can be raised to corresponding author and will be shared based on reasonable request.
